# Cell-Phone Addiction: A Review

**DOI:** 10.3389/fpsyt.2016.00175

**Published:** 2016-10-24

**Authors:** José De-Sola Gutiérrez, Fernando Rodríguez de Fonseca, Gabriel Rubio

**Affiliations:** ^1^Department of Psychobiology, Psychology Faculty, Complutense University of Madrid (Universidad Complutense de Madrid), Madrid, Spain; ^2^Clinical Management of Mental Health Unit, Biomedical Research Institute of Málaga, Regional University Hospital of Málaga (Unidad de Gestión Clínica de Salud Mental, Hospital Regional Universitario de Málaga, Instituto de Investigación Biomédica de Málaga – IBIMA), Málaga, Spain; ^3^Istituto de Investigación i+12, Hospital Universitario 12 de Octubre de Madrid, Madrid, Spain

**Keywords:** addiction, behavioral addiction, cell-phone addiction, dependence, internet addiction

## Abstract

We present a review of the studies that have been published about addiction to cell phones. We analyze the concept of cell-phone addiction as well as its prevalence, study methodologies, psychological features, and associated psychiatric comorbidities. Research in this field has generally evolved from a global view of the cell phone as a device to its analysis *via* applications and contents. The diversity of criteria and methodological approaches that have been used is notable, as is a certain lack of conceptual delimitation that has resulted in a broad spread of prevalent data. There is a consensus about the existence of cell-phone addiction, but the delimitation and criteria used by various researchers vary. Cell-phone addiction shows a distinct user profile that differentiates it from Internet addiction. Without evidence pointing to the influence of cultural level and socioeconomic status, the pattern of abuse is greatest among young people, primarily females. Intercultural and geographical differences have not been sufficiently studied. The problematic use of cell phones has been associated with personality variables, such as extraversion, neuroticism, self-esteem, impulsivity, self-identity, and self-image. Similarly, sleep disturbance, anxiety, stress, and, to a lesser extent, depression, which are also associated with Internet abuse, have been associated with problematic cell-phone use. In addition, the present review reveals the coexistence relationship between problematic cell-phone use and substance use such as tobacco and alcohol.

## Introduction

Since the appearance of the cell phone, the anomalous use of this device has called into question whether the abuse of its use could lead to addiction. This problem is identical to the one regarding the existence of behavioral addictions as opposed to substance addictions (1). The existence of cell-phone addiction, as opposed to it being the manifestation of an impulsivity disorder, has been questioned without necessarily considering the concept of addiction (2, 3). To date, the DSM-5 has only recognized compulsive gambling as a behavioral addiction, considering the rest of these types of abuse as impulse disorders, and the clinical world has not done much more than proclaim that many of them are true addictions that affect patients’ lives.

Prior to the arrival of the cell phone, abundant research had been conducted on behavioral addictions to videogames (4), exercise (5), online sex (6), food (7), shopping (8, 9), work (10), and the Internet (11–15). Indeed, for several authors, a large number of behaviors are potentially addictive (16) if there is a concurrence of negative consequences and physical and psychological reinforcements in a specific context (17).

Before reviewing the characteristics of cell-phone addiction, it is important to highlight the uniqueness of behavioral addiction in relation to drug or substance addiction. In substance addiction, with the exception of alcohol that shows a more dimensional course profile, there is a clear moment at which changes in and interferences with daily life can be observed. In the case of behavior, it is difficult to determine whether problems result from problematic behavior, personality traits, or psychiatric comorbidities. However, the existence of an underlying biological sub-layer, which can manifest itself through pharmacological procedures, is indubitable. Thus, administering specific dopamine agonists can activate previously non-existent behaviors, such as compulsive gambling, compulsive eating, hypersexuality, and compulsive shopping (18–21).

An increasing number of studies have focused on the most important body of behavioral addictions today – the Internet, videogames, and cell phones. Historically, Internet use could present as either a global addiction or interaction with addictive contents and activities. In this sense, Young (12) studied five different forms of addictive behavior on the Internet: (1) the computer itself, (2) the search for information, (3) interaction compulsions, including contact with the web through online games, shopping, etc., (4) cybersexuality, and (5) cybercontacts. Subsequently, Young solely studied games, online sexual contacts, and text messaging (14).

If the Internet was initially the technological addiction par excellence, the cell phone soon emerged as a source of potentially addictive behavior, particularly since the arrival of smartphone devices (22, 23), along with the evolution from a global approach to a progressive differentiation of addictions by contents and concrete applications. Whether the problem is the cell phone itself or its contents and applications (24) is a topic of current debate, similar to previous debates with respect to the Internet (25, 26).

From this perspective, the cell phone offers activities that can lead to problematic use (3, 27). There is evidence that the smartphone, with its breadth of applications and uses, tends to induce greater abuse than regular cell phones (28).

In general, Brown (29) and Griffiths (17, 30) note that an addiction entails abuse without control, alterations in mood, tolerance, abstinence, and personal harm or conflicts in the environment, as well as a tendency to relapse. Sussman and Sussman (31) profile addiction, in its broadest sense, as the capacity to get “hooked” on reinforcing behaviors, excessive worry about consumption or behaviors with high positive reinforcement, tolerance, loss of control, and difficulty in avoiding said behavior, despite its negative consequences. Specifically, Echeburua et al. (32) noted as defining elements of behavioral addictions the loss of control, the establishment of a dependent relationship, tolerance, the need for progressively more time and dedication, and severe interference with daily life. Cía (33) highlights the automatism by which these behaviors lead to uncontrollable use, in addition to feelings of intense desire or irresistible need, loss of control, inattention to usual activities, the focalization of interests on the behavior or activity of interest, the persistence of the behavior despite its negative effects, and the irritability and malaise associated with abstinence.

Following the criteria of Hooper and Zhou (34), O’Guinn and Faber (8), and Hanley and Wilhelm (35) regarding motivations of use, Shambare et al. (36) consider cell-phone addiction to be one of the greatest addictions of the current century. They highlight six types of behavior, habitual (habits performed with little mental awareness), mandatory (officially required o parentally mandated), voluntary (reasoned and conducted for specific motivations), dependent (motivated by the attached importance of social norms), compulsive (strong urge to continuously perform the behavior), and addictive, or behavior defined by the user’s progressive exclusion of other activities, causing physical, mental, and social harm, while attempting to control the user’s dysphoric feelings. Therefore, excessive attention and uncontrolled dedication to one’s cell phone is an addiction.

In any case, the research and literature on Internet, videogame, and cell-phone use are ever-increasing. One bibliometric study (37) indicated a progressive and growing body of research, with the Internet being the most highly studied area, followed by videogames and then cell phones. In recent years, research interest in cell-phone use has notably increased.

## Cell-Phone Addiction

In April 2015, the number of cell-phone lines exceeded 53.6 million in Spain, which was1.4% higher than that of the previous year, with a penetration of 108.5% [National Commission of Markets and Competence (38)]. This amounts to slightly greater than one cell phone per person, and 81% of these cell-phone lines were associated with smartphones in 2014 [Telephonic Foundation (39)]. The age of cell phone initiation is becoming increasingly younger: 30% of 10-year-old Spanish children have a cell phone; the rate increases to nearly 70% at age 12 and 83% at age 14. Furthermore, starting at the age of 2–3 years, Spanish children habitually access their parents’ devices (40).

These data imply that the cell phone enables behavioral problems and disorders, particularly in adolescents. This fact has become more and more evident in communications media, inspiring new pathologies, such as “Nomophobia” (No-Mobile-Phobia), “FOMO” (Fear Of Missing Out) – the fear of being without a cell phone, disconnected or off the Internet, “Textaphrenia” and “Ringxiety” – the false sensation of having received a text message or call that leads to constantly checking the device, and “Textiety” – the anxiety of receiving and responding immediately to text messages (28).

Physical and psychological problems have reportedly resulted from cell-phone abuse, including rigidity and muscle pain, ocular afflictions resulting from Computer Vision Syndrome reflected in fatigue, dryness, blurry vision, irritation, or ocular redness (41), auditory and tactile illusions – the sensation of having heard a ring or felt a vibration of a cell phone (42, 43), and pain and weakness in the thumbs and wrists leading to an increased number of cases of de Quervain’s tenosynovitis (44).

In broader behavioral terms, the following problematic manifestations have also been noted, frequently compared to and corroborated by the diagnostic criteria of the DSM (see Table 1):

– Problematic and conscious use in dangerous situations or prohibited contexts (45) with social and familial conflicts and confrontations, as well as loss of interest in other activities (46–49). A continuation of the behavior is observed despite the negative effects or the personal malaise caused (50, 51).
– Harm, repeated physical, mental, social, work, or familial interruptions, preferring the cell phone to personal contact (52–54); frequent and constant consultations in brief periods (3) with insomnia and sleep disturbances (55, 56).
– Excessive use, urgency, abstinence, tolerance, dependence, difficulty controlling, craving, increasing use to achieve satisfaction or relaxation or to counteract a dysphoric mood (34, 57, 58), the need to be connected, feelings of irritability or of being lost if separated from the phone or of sending and viewing messages with feelings of unease when unable to use it (54, 59–61).
– Anxiety and loneliness when unable to send a message or receive an immediate response (62); stress and changes in mood due to the need to respond immediately to messages (55, 63).

**Table 1:** **Symptomatology of problematic cell-phone use vs. DSM-5 criteria for compulsive gambling and substance use**.

Symptomatology of problematic cell-phone use, according to the references noted in this section	DSM-5 Criteria–Substance use disorder (64)	DSM-5 Criteria for compulsive gambling – Gambling disorder (64)
Problems and conscious use in dangerous situations or in prohibited contexts	Dangerous use	Turns to loans when faced with the desperate personal economic situation produced by gambling
	Difficulty performing important social, work or leisure activities due to use	
Social and family conflicts and confrontations, as well as loss of interest in other activities	Social, interpersonal problems related to use	Personal and social relationships, jobs, studies, or careers are in danger or are lost
	Abandonment of usual activities due to use	
Continuing behaviour despite the negative effects and/or personal malaise it causes	Continues using the substance despite being conscious of recurring or persistent psychological or physical problems, which appear to be caused or exacerbated by substance use	Even when losing money, keeps gambling
Harm, physical, mental, social, work, or family disturbances		
Difficulty of controlling	Repeated attempts to quit, to stop using	Repeated unsuccessful efforts to avoid said conduct
Frequent and constant checking of phone in very brief periods of time with insomnia and sleep disturbances	Spends a lot of time getting the substance, using it, or recovering from its effects	Excessive preoccupation about gambling (persistent thoughts, memories of previous experiences, search for new opportunities to gamble, means to get money and continue gambling)
Tolerance	Tolerance	Growing need to gamble a progressively increasing amount of money in order to achieve well-being
Increase in use to achieve satisfaction or relaxation or to counteract a dysphoric mood		
Excessive use, urgency, need to be connected	Progressive increase in use	Search for gambling opportunities when faced with feelings of unease, such as anxiety, guild, depression, powerlessness, etc
Need to respond immediately to messages, preferring the cell phone to personal contact		
Abstinence, dependence, craving	Abstinence syndrome	Lies to self, denies dependence
Anxiety, irritability if cell phone is not accessible, feelings of unease when unable to use it		Unease and irritability when trying to avoid or stop said behaviour

Chóliz (65), supporting his theory using the DSM-IV-TR for substance addiction, mentioned four factors that define addiction and dependence in students: *abstinence, lack of control, tolerance*, and *abuse and interference with other activities* (59, 66). Similarly, in a recent longitudinal study on student smartphone use, addictive behavior was related to the downloading and use of specific applications along with compulsive consultation and writing. That is, a non-addicted user can spend the identical amount of time on the cell phone as an addicted user, but the non-addicted user’s time is constant, more focused on concrete tasks and less disperse (3).

There exists, however, a broad spectrum of positions taken by researchers, ranging from the absolute existence of addiction to a broader interpretation of these symptoms, as the result of an impulse control disorder or of problematic or psychopathological personality traits, which offer a greater range of behavioral possibilities beyond addiction itself. In this sense, Sansone and Sansone (55) note that the delineations between abuse, misuse, dependence, and addiction have yet to be clearly defined. Toda et al. (67) note that cell-phone abuse can also be seen as a behavior congruent with a certain lifestyle.

However, considering the general profiles of addiction indicated, the symptoms and specific predicament observed, and analyzing its correspondence to the criteria for pathological gambling in the DSM-5 and substance addiction – a fundamental comparative medium for many researchers evaluating phone addiction – an important parallelism can be appreciated, which requires the consideration of its existence without excluding other potentially problematic behaviors.

Finally, there is a known vulnerability or “breeding ground” associated with the development of substance addiction in general, and for behavioral addictions in particular, that is defined by low self-esteem, difficulty with conflict, impulsivity and sensation seeking, intolerance of pain and sadness, and/or a tendency toward depressive or dysphoric states (33). This could explain the frequent coexistence of problematic cell-phone behavior and problematic traits or psychiatric comorbidities, as seen below.

## Prevalence

Sizeable prevalence data (see Table 2) have been generated in response to specific addiction criteria, dependence, problematic use, excessive use, and risky behavior. Within each criterion, broad percentage ranges are supported by various methodologies, instruments, and samples, making comparisons difficult.

**Table 2:** **Prevalence data**.

	Scale	Sample/area	Age/population	Prevalence (%)	Criterion
Beranuy Fargues et al. (68)	CERM	430 + 209/Barcelona	13–18	5.35	Addiction
			19–25	5.26	
Toda et al. (67)	MPDQ	271/Japan	19–23	18.8	Dependence
				17.5	
Jenaro et al. (69)	COS	337/Salamanca	18–32	10.4	Addiction
Perry and Lee (70)	*Ad Hoc*	214/Mauricio	19–25	6–11	Addiction
Leung (57)	MPAI	624/Amsterdam	14–28	28.7	Addiction
Addiction Institute [Instituto De Adicciones (71)]	*Ad Hoc*	556/Spain	12–25	8.5	Problematic use
				0.4	
Leung (72)	MPAI	402/Hong Kong	14–20	27.4	Addiction
Ha et al. (62)	ECPUS	595/Korea	15.9 mean	33	Excessive use
Koo (73)	CPAS	577/Korea	Adolescents	2.9	Addiction
				8.2	Problematic use
Sanchez Martinez and Otero (74)	*Ad Hoc*	1328/Madrid	13–20	20	Dependence
Beranuy Fargues et al. (75)	CERM	1.879/Barcelona	15–25	5.57	Abuse
					Addiction
Koo (76)	CPAS	469/Korea	High school students	4.1	Addiction
				7.5	Abuse
Halayem et al. (77)	STDS	120/Tunisia	13–20	33	Addiction
					Dependence
Ruiz-Olivares et al. (78)	*Ad Hoc*	1011/Córdoba	18–29	32.6	Problematic use
Lu et al. (79)	STDS	146/Japan	22–59	3.1	Addiction
				5.4	Dependence
Martinotti et al. (80)	MAT	279/Italy	13–20	6.3	Problematic use
					Addiction
Lopez-Fernandez et al. (81)	MPPUS	1.132/Spain	12–18	14.8	Problematic use
				5.4	At risk
Lopez-Fernandez et al. (82)	MPPUS	1.026/England	11–18	10	Problematic use
				10.5	At risk
Mazaheri and Najarkolaei (83)	MPAI	1180/Iran	18–39	64.5	Addiction
				56.2	
Tavakolizadeh et al. (84)	MPAI	700/Iran	University students	36.7	Addiction
Shin (85)	MIUI	597/Korea and USA	University students	8.88	Dependence
Kalhori et al. (86)	MPPUS	600/Tehran	20–30	23.4	Problematic use
					Dependence
Tosell et al. (3)	Longitudinal online registry	34/USA	University students	62	Addiction
	SAMI/CPAS				

It is known that self-reported questionnaires differ in self-implication and sincerity depending on whether they are administered in person or by correspondence. In fact, certain behaviors tend to be minimized in self-reports (105). Taking into account that several studies on cell-phone addiction have used the self-attribution or self-perception of the interviewee (89), Beranuy Fargues et al. (68) observed that in this sense, 22.1% of adolescents and 27.9% of young people were considered to be cell-phone addicts, although only 5.35% and 5.26% of them exhibited dangerous or harmful behaviors. Billieux et al. (45) also found that certain dimensions of impulsivity, such as impatience, low perseverance, and length of cell-phone possession, were predictors of greater self-attribution of addiction.

Therefore, self-attribution results in high prevalence data and leads to a greater subjective sensation of addiction, which is decreased when using objective or validated criteria beyond subjective self-perception (50).

The prevalence samples are generally based on young students and adolescents, which means that prevalence essentially refers to this population without the consistent availability of exact ages. Although we know that cell-phone abuse can be truly problematic in young students and adolescents, we lack a broader understanding of the problem with respect to the general population. It is important to evaluate the differences between the adolescent and adult populations and observe the effects of cell-phone use on each of them (106). In addition, relevant inter-geographical and intercultural differences have not been sufficiently studied to date, although some studies have noted a greater prevalence in Middle East (Iran) and East Asian populations, specifically in Korea where university students showed a greater level of dependence (11.15%) than the Americans (6.36%) (85).

## Methodological Problems with the Study of Cell-Phone Addiction

Methodology and evaluation instruments (see Table 3) are determined by their base criteria of origin. Essentially, there is one line of inquiry that considers addiction to be an extensive concept, not limited to substances, that has a foundation in its neurobiologic basis (1, 107, 108). This concept has been used in the criteria of pathological gambling (26, 57, 72, 75) and substance addiction [Yen et al. (90), Chóliz and Villanueva (66), Chóliz and Villanueva (61), Chóliz (54), Labrador Encinas and Villadangos González (49), Merlo et al. (98), Kwon et al. (60), Roberts et al. (27), and among others]. Some authors have based their research on the criteria of Internet addiction or general behavioral addiction, which had a clear support on the criteria established from substance abuse research (34, 80, 85, 88, 91, 95, 96, 102).

**Table 3:** **Instruments and methodologies**.

Instrument	Items	Sample	Base criterion	Construct	Dimensions/factors	Reliability (α)
Cellular Phone Dependence Questionnaire (CPDQ) (87)	20 items, Likert scale (0–3)	University students	–	Dependence	6 factors *via* AFE	0.86
Mobile Phone Dependence Questionnaire (MPDQ) (67)	20 items, Likert scale (0–3)	University students	–	Problematic use, dependence	1 factor	0.86
Mobile Phone Problem Use Scale (MPPUS) (2)	27 items, Likert scale (0–10)	Adults	–	Problematic use		0.93
Cell Phone Over-Use Scale (COS) (69)	23 items, Likert scale (1–6)	Grade school students	–	Addiction		0.87
				Excessive use		
SMS Problem Use Diagnostic Questionnaire (SMS-PUDQ) (88)	8 items, Likert scale	Grade school students	Based on the criteria of Young (13) for Internet addiction	Excessive use	2 factors *via* AFE	0.84–0.87
				pathological use		
				SMS		
Mobile Phone Usage Scale (MPUS) (34)	33 items, Likert scale (1–5)	University students	Based on the criteria for use and addictive shopping (8)	Dependence	6 use factors validated *via* AF	Factor analysis
				Addiction		
				Habitual use		
				Mandatory use		From 0.53 to 0.88
				Voluntary use		
				Compulsive use		
Mobile Phone Addiction Index (MPAI) (57, 72)	17 items, Likert scale (1–5)	Adults, adolescents	Based on criteria for pathological gambling	Addiction	4 factors *via* AFE	0.90
Problematic Mobile Phone Use Questionnaire (PMPUQ) (45)	30 items, Likert scale (1–4), plus 1 dichotomous item	Adults	–	Prohibited use	4 dimensions	Scales 0.65 < α < 0.85
				Dangerous use		
				Dependence		
				Economic problems		
Excessive Cellular Phone Use Survey (ECPUS) (62).	20 items	Adolescents	–	Excessive use		0.87
Text-message Dependency Scale (TMDS) o Self-perception of Text-message Dependency Scale (STDS) (89)	15 items, Likert scale (1–5)	Grade school students	–	Self-perception of dependence and addiction to SMS	3 factors	
Questionnaire of Experiences related to the Cell (*Cuestionario de Experiencias relacionadas con el móvil – CERM*) (75)	10 items, Likert scale (1–4)	Young and adolescent students.	Criteria for substance addiction and pathological gambling.	Abuse	2 factors	0.80
				Addiction		
Test of Mobile Phone Dependence (TMP) (54, 61, 66)	38 items, Likert scale	Adolescents	DSM-IV-TR criteria for Substance Abuse disorders	Addiction, dependence	3 factors	Scales 0.85 < α < 0.91
Cell-Phone Addiction Scale for Korean Adolescents (CPAS) (73)	20 items, Likert scale	Adolescents	–	Addiction	3 factors *via* AFE	0.92
				Excessive use		
Problem Cellular Phone Use Questionnaire (PCPU-Q) (90)	12 items, dichotomous scale	Adolescents	DSM-IV-TR criteria for Substance Abuse disorders	Problematic use	Symptomatology of problematic use	0.85
Questionnaire to Detect New Addictions (*Cuestionario de Detección de Nuevas Adicciones – DENA*) (49)	12 items, 8 with Likert scale (0–3)	Adolescents	DSM-IV-TR criteria for Substance Abuse disorders	Addiction, abuse		
Mobile Phone Involvement Questionnaire (MPIQ) (91)	8 items, Likert scale (1–7)	Adolescents and youths	Criteria for behavioral addiction from Brown (92)	Addiction	1 dimension or factor	0.78
			Substance abuse criteria			
Mobile Addiction Test (MAT) (80)	10 items, Likert scale (1–3)	Grade school students	Comparison with other behavioral addictions	Addiction		
				Problematic use		
Mobile Phone Usage Behavior Scale (MPUB) (93)	4 open questions	Students	–	Frequency of use/day	No. of calls made	0.68
					No. of calls received	
					No. of messages read	
					No. of messages received	
Cell-Phone Addiction Assessment Questionnaire (KBUTK) (26)	33 items, Likert scale (1–5)	Grade school and university students	Pathological gambling criteria	Addiction	4 factors of addiction	0.91
Test Messaging Gratification Scale (TMG) (94)	47 items, Likert scale (1–7)	Grade school students	–	Gratification with SMS	7 factors *via* AF	0.86
Bergen Facebook Addiction Scale (BFAS) (95)	18 items, Likert scale (1–5)	University students	Standard addiction criteria from the literature	Facebook addiction	1 factor *via* AF	0.83
Mobile Phone Addiction Scale (MPAS) (96)	11 items, Likert scale (1–6)	Female university students	Based on the Internet addiction scaleby Young (97)	Addiction	3 factors	0.86
Problematic Use of Mobile Phones (PUMP) Scale (98)	20 items, Likert scale (1–5)	Adults (18–75 years)	DSM-IV-TR criteria for Substance Abuse disorders	Problematic use	1 factor	0.94
				Addiction		
Smartphone Addiction Scale (SAS) (60)	48 items, Likert scale (1–6)	Adults (18–53 years)	DSM-IV-TR criteria for Substance Abuse disorders	Smartphone	6 factors *via* AFE	0.97
				Addiction		
Mobile Phone Use Questionnaire (MP-UQ) (99)	29 items	Patients with anxiety and agoraphobia	DSM-IV-TR criteria.	Nomophobia		
Manolis/Roberts Cell-Phone Addiction Scale (MRCPAS) (27)	4 items	Grade school students	DSM-IV-TR criteria for Substance Abuse disorders	Craving	1 factor	>0.70
				Addiction		
Mobile Phone Activities and Addiction of Parents (MPAA) (100)	21 items	Parents of students	–	Applications, use of cell-phone contents, and addiction among parents	7 factors of activities and addiction	0.91
Mobile Internet Usage Index (MIUI) (85)	19 items, dichotomous response	University students (USA and Korea)	Adaptation of the IAT (Internet Addiction Test) (101)	Cell Internet dependence		
Smartphone Addiction Inventory (SPAI) (102)	26 items, Likert scale (1–4)	University students	Adaptation of the Chen Internet Addiction Scale (CIAS) (103)	Smartphone addiction	4 factors *via* AF	0.94
Smartphone Addiction Questionnaire (SPAQ) (104)	5 open questions	University students	Adaptation of the SAS (60)	Frequency of use/day	Frequency of use	0.76
	34 items			Addiction	Addiction to activities and applications	
					Symptoms of addiction	
Smartphone Addiction Measurement Instrument (SAMI) (3)	15 items, Likert scale (1–5)	University students		Addiction		

Another line of research accepts the concept of cell-phone addiction, broadening the possibilities and defining the behavior, together with the term “addiction” related to compulsive behavior (109), dependent behavior (34, 45, 67, 85, 87), and problematic, excessive, or pathological use (62, 80, 88), which leads to evaluation instruments with relatively broad behavioral ranges. This research line is characterized by an emphasis on the coexistence of lack of impulse control and addiction. From this perspective, lack of control is the result of, or coexists with, other pathologies in which impulsivity plays a relevant role (110, 111). Therefore, the fact that cell-phone use is reinforcing could lead to problematic behaviors without necessarily needing to label them as addictions (2, 3, 69).

Methodologically, the majority of these studies are cross-sectional and based on questionnaires using students and samples of convenience that typically contain only one sample point, although several recent studies have been based on longitudinal telematic registries. Currently, the following lines of inquiry are most salient:

– Research using questionnaires based on self-described addiction [Beranuy Fargues et al. (68); Chen (112); Perry and Lee (70); Halayem et al. (77); Hashem (113), among others] – the concept of addictionis presupposed from the start, and a personal self-evaluation is requested from the interviewee. They generally produce high prevalence data, as mentioned earlier.
– Research using questionnaires about problematic behaviors, classifying users as a function of their use (2, 45, 62, 69, 90) without necessarily addressing the concept of addiction – addiction in this case is validated by external criteria, such as the DSM-IV-TR or DSM-5, taking dangerous, problematic, or dependent use as behaviors into account [Hooper and Zhou (34), Leung (57), Leung (72), Igarashi et al. (89), Chóliz and Villanueva (66), Chóliz and Villanueva (61), Chóliz (54), Koo (73), Walsh et al. (91), Martinotti et al. (80), Pawlowska and Potembska (26), Merlo et al. (98), Kwon et al. (60), and among others].
– Longitudinal studies with behavior-registering devices using software installed on the cell phones of the participants where the specific use of each participant was registered continuously – this is the most recent methodology, and relatively small samples are used to register content, usage time, and frequency of consultation. One such study showed that the total perceived usage time reported on the questionnaires was higher than the actual registered data (3, 23, 114, 115), meaning that the self-perception of the time dedicated to the content reported in the questionnaires was less than the actual time registered by the application, indicating a clear underestimation of usage (115).
– Qualitative studies that seek the direct experience of the users (109, 116, 117) – these are based on personal and group interviews, offering direct information that is very useful for the design of quantitative research instruments, as well as for the evaluation and analysis of the results obtained.

In general, these instruments and studies have evolved from the study of global cell-phone use behavior toward specific behaviors, such as using smartphones (60, 102), mobile Internet (85), social networks in general (27, 118, 119), Facebook in particular (27, 95), text messages (88, 89), and WhatsApp (63) or the consequences of such behaviors, i.e., nomophobia (99). Therefore, in addition to the study of the behavior associated with the device itself, relevance is given to its use and differentiation *via* specific activities, applications, and consequences. In this sense, Lin et al. (102) suggest that the smartphone may have given rise to a new type of addictive behavior defined as a multidimensional construct, as well as for the Internet addiction.

## Sociodemographic Differences

There is great diversity in the data and studies on problematic cell-phone use, although the majority of them essentially analyze age and sex differences, with the evaluation of educational level and economic status being more or less conclusive. Although the studies we reviewed had very diverse geographical origins, an analysis of cultural geographical diversity is lacking in the literature.

### Differences by Age

The youngest group, particularly adolescents, is the most highly affected by and at risk for both substance and behavioral addiction (120), which has led the majority of studies to address these age groups.

In general, the data show that the total time spent on cell phones decreases with age, with the highest times reported for people less than 20 years old, principally adolescents, approximately 14 years old (50, 61, 75, 78, 82, 83, 121). This fact is related to the decreased self-control found in this age group (2). Specifically, the most frequent use of their time is spent on text messaging (22, 58, 79), with other forms of contact increasing over time (122).

Cell-phone use in adolescents is so important that some adolescents never turn off their cell phones at night, fostering vigilance behavior that makes rest difficult (59). Specifically, 27% of young people between 11 and 14 years of age admit that they never turn off their cell phones, a behavior that increases with age such that between 13–14 years old, one out of every three young people never turns off his/her device (40).

The age of possession of one’s first cell phone is also relevant: the younger age at which this occurs, the greater the probability of problematic use in the future. In particular, Sahin et al. (56) found that the greatest indices of problematic use or addiction are found when one’s first phone is obtained at an age younger than 13 years.

### Differences by Gender

Virtually all the studies indicate that females have higher levels of dependence and problematic use than males (69, 74, 75, 81). Female cell-phone use is typically related to sociability (2), interpersonal relationships and the creation, and maintenance of contacts and indirect communication, and texting and instant messaging are their most frequently used applications (67, 122). In addition, a cell phone can be used to avoid unpleasant moods (59, 61), which leads to impatient and uneasy behavior associated with conscious self-control and spending difficulties (49, 78).

For males, cell-phone use is simultaneously based on text messages, voice conversations (45, 123), and gaming applications (24, 124), and they show a higher tendency than females to use their cell phone in risky situations (45). A study carried out by Roberts et al. (27) found that the most problematic applications are voice calls, text messages, and social networks. The differences between males and females are based on usage time rather than utilization. Females spend more time than males on each of these applications, which leads to behavior oriented toward intense and close social relationships, whereas males use their time in a more practical and instrumental way.

For females, therefore, the cell phone is a means of social contact, in which messaging and social networks play a relevant role, while for males, a more diversified type of usage is observed. This differs from Internet use, which shows the inverse profile: problematic behavior is observed more frequently in males (125). Cell-phone abuse thus responds to a pattern of greater lack of impulse control (126); similarly, being female could be a protective factor for problematic Internet use (78).

### Education, Cultural Level, and Economic Status Differences

Despite the lack of evidence of educational and economic level differences in usage (127), Mazaheri and Najarkolaei (83) found that students from families with higher cultural and economic levels have higher levels of dependence, a fact they relate to the isolation and loneliness felt when studying far from home; here, the cell phone is a tool for contact. In the same sense, Tavakolizadeh et al. (84) confirmed a direct relationship between education level and problematic use, which they attributed to the time spent away from home and the isolation caused by extended periods of study. Sanchez Martinez and Otero (74) confirmed a relationship between students and problematic cell-phone use, negative family relationships, and parents with a high level of education without economic difficulties. They explain that this relationship is due to the need to maintain compensatory social relationships.

Sahin et al. (56), on the contrary, found that the level of cell-phone addiction is greater in students from families with lower versus higher incomes. Lopez-Fernandez et al. (81) also observed a significant relationship between student cell-phone use and their parents’ level of education. The higher the level of education of the father or mother was, the less problematic the cell-phone use; if the parents had university degrees, the exclusive technological entertainment of their children decreased. In the same direction, Leung (57) found a relationship between low socioeconomic and educational levels and problematic cell-phone use.

In terms of family education, Zhou et al. (100) also observed a significant relationship between parents’ abuse of and dependence on cell phones and children’s addiction to the Internet and other technologies, which they interpreted as the result of affective abandonment.

### Geographical and Cultural Differences

It is logical to assume that geographical and cultural differences exist regarding problematic cell-phone use; however, scarce conclusive geographical data are available on the topic. It appears that greater cell-phone dependence exists in East Asian countries, such as Korea, which can be explained by their sizeable cell-phone offerings and high technological penetration among the youngest strata. Shin (85) carried out a comparative study evaluating the degree of mobile Internet dependence of university students in the United States and Korea. Their data confirmed that the Koreans showed a greater level of dependence (11.15%) than the Americans (6.36%).

## Personality and Psychological Variables

Essentially, problematic cell-phone studies aim to detect the variables or personality traits that coexist with problematic or addictive behavior. In this sense, one can also talk about vulnerability, insofar as some of these traits can be precursors to or predictors of addiction to either drugs or certain behaviors (33).Specifically, they have focused on the five-factor model (FFM) of personality as well as on self-esteem, self-concept, self-identity, and impulsivity.

### Five-Factor Model

The “Big Five PersonalityTraits,” also known as the FFM, has been used in research on both cell phone and substance addiction (128).The FFM establishes five dimensions of personality (extraversion, openness to experience or change, conscientiousness, agreeableness, and neuroticism or emotional instability).

Takao (129), using the NEO five-factor inventory (130), observed that being female, extrovert, neurotic, and low opened to experience predict 13.5% of cases of problematic cell-phone use. Neuroticism is related to low self-esteem and the need for social approval, while low openness to experience implies a tendency to avoid disagreeable emotional states.

Kuss and Griffiths (118) found that extraverts use social networks to make and improve contacts, whereas introverts use them to compensate for their difficulties in relating to people. Both extraverts and introverts are potential addicts, particularly extraverts with low scores in conscientiousness and introverts with high scores in neuroticism and narcissism. Giota and Kleftaras (119) observed that the problematic use of social networks is related to neuroticism and agreeableness as well as to depression, particularly in females.

Lane and Manner (22) confirmed that extraversion is a potent predictor of smartphone possession, with text messages and instant messaging being the most frequently used applications. At the same time, a high agreeableness score predicts higher phone calling than texting, which suggests that social contact is supported by direct communication.

Similarly, Bianchi and Phillips (2) studied problematic cell-phone use as a function of age, extraversion, and low self-esteem. Specifically, extraversion was associated with the need for more frequent self-stimulation *via* texts than direct contact. In their study, neuroticism was not a predictive variable; however, they observed that low self-esteem predicted problematic use insofar as it determined an indirect messaging style of communication. Notably, self-esteem can change according to context and time and can be considered to be a state (131) that is amenable to contextual cell-phone use (127). This suggests that problematic cell-phone use related to low self-esteem might be situational in nature.

Igarashi et al. (89) studied the problematic use of text messages vis-à-vis direct personal relationships. They found that dependence and excessive use are explained, on the one hand, by extraversion, which reflects the need and desire to maintain communication with others and establish new relationships, while on the other hand, text messaging to address a need for security and compensate for the fear of social loss can be explained by neuroticism.

Andreassen et al. (95) focused their study on Facebook to develop the Bergen Facebook Addiction Scale (BFAS). They found that the BFAS is positively correlated not only with the Addictive Tendencies Scale (132) but also with neuroticism and extraversion and is negatively correlated with conscientiousness. Two perspectives can be appreciated here: extraversion maintains a direct relationship with problematic cell-phone use, whereas this relationship is inverse with respect to the Internet (133). Thus, Facebook can be addictive, and the extraversion profile can be either direct or inverse, depending on whether Facebook is used *via* a cell phone or computer.

In general, the abuse of sending text messages is associated with a strong tendency for extraversion and low self-esteem. In social networks, in addition to extraversion, neuroticism is a likely factor because individuals with high levels of anxiety and insecurity can use social networks for support and security (134). Comparatively, the use of social media on a computer reflects a tendency for evasion, social phobia, shyness, introversion, neuroticism, low levels of self-esteem, and self-sufficiency, in addition to sensation seeking (135).

### Impulsivity and Sensation Seeking

Impulsivity is another traditionally considered predictive dimension of cell-phone abuse, and we have previously analyzed its role as a precursor or vulnerability factor for behavioral addictions (136, 137). In particular, Billieux et al. (45) analyzed the role of impulsivity according to the four components of the UPPS [Urgency, (lack of) Premeditation, (lack of) Perseverance and Sensation seeking] reference scale (138). They found that urgency, lack of premeditation, and lack of perseverance are inversely related to self-control. However, urgency, defined as the tendency to experience strong impulses that cannot be postponed as a result of negative affective states, is the component that best predicts problematic cell-phone use. Thus, a high urgency score relates to an increased number of calls, duration, and number of text messages sent. Urgency is similarly related to inadequate strategies for emotional self-regulation, such as ruminating thoughts that provoke and maintain negative affective states. Problematic cell-phone use in this case reflects an attempt to control these negative emotional states. On the other hand, lack of perseverance can be reflected in the number and duration of cell-phone calls as well as in associated economic problems, while lack of premeditation entails its use in dangerous or prohibited situations, which is related to sensation seeking (127).

Sensation seeking is a personality trait that entails the dimensions of thrill and adventure seeking, lack of inhibition, experience seeking, and sensitivity to boredom (139, 140). It is characterized by the need for new experiences that are uncommon, varied, and intense, with accompanying physical, social, legal, and/or financial risks, and frequently coexists with impulsivity in addictive behavior (141). Previous studies have found a relationship between leisure boredom and self-esteem; Leung (57, 72) confirmed that boredom, measured by the Leisure Boredom Scale (142), sensation seeking, using the Adventure subscale (143), and self-esteem through the Rosenberg Self-esteem Scale (144) are significant predictors of problematic cell-phone use.

### Self-esteem, Self-identity, Self-control, and Social Environment

Concepts such as self-esteem, self-control or social self-vigilance, and dependence on the environment are found in the majority of studies on problematic cell-phone use. Takao et al. (145) observed that problematic cell-phone use is a function of the need for social approval and self-control but is unrelated to loneliness. The latter, on the contrary, is related to Internet abuse (146). Given that loneliness coexists with introversion, it can be concluded that differentially, these variables are predictors of Internet addiction but not necessarily cell-phone addiction. Nevertheless, Bhardwaj and Ashok (147) found a correlation between cell-phone addiction and loneliness. The need for social approval, expressed in the time dedicated to writing and reading messages, has also been associated with low self-esteem (148).

Park et al. (149) found that imitation of others, low self-esteem, and social anxiety contributed to cell-phone abuse. However, as in other studies, it is not necessarily voice conversations but rather the number of text messages that is frequently the result of problematic use.

Walsh et al. (91) differentiated the frequency of cell-phone use from personal implication or dependence as measured by the Mobile Phone Involvement Questionnaire (MPIQ). They considered that self-identity, or the perceived value of the cell phone for self-concept and others’ approval, would be a predictor of frequency of use, while self-identity and others’ approval would determine dependence or implication. That is, they considered cell-phone dependence to be related to dependence on the social environment. Later, Walsh et al. (93) found that self-identity at an early age predicts frequency of use, while dependence or personal implication with the cell phone maintains important relationships with being female, youth, self-identity, and group norms.

Similarly, self-esteem is a commonly examined trait in problematic cell-phone use studies. Cell-phone abuse and addiction has even been explained using Attachment Theory (150), which establishes that new-borns, from birth, must develop a close relationship with at least one principal caretaker in synchrony with their needs and emotional states for healthy social and emotional development. There is evidence that insecure attachment styles are associated with low self-esteem (151, 152) and, therefore, potential predictors of problematic cell-phone use (127).

Finally, Billieux (127) summarized the current open lines of inquiry, indicating four groups in the problematic cell-phone use research: (a) impulsivity, from its limited capacity for self-control and emotional regulation, (b) relationship maintenance, which portrays cell-phone abuse as a means to obtain security in affective relationships and is characterized by low self-esteem and high levels of neuroticism, (c) extraversion, which associates excessive use with sociability and the intense desire to maintain relationships, and (d) Cyberaddiction in consonance with smartphone technology, which allows access to diverse utilities and applications online. The latter explains abusive use as a result of this technological environment’s attraction. From this viewpoint, addiction could lead to other harmful behaviors, such as Internet or videogame abuse.

## Psychological Problems and Psychiatric Comorbidities

With respect to the psychological problems derived from cell-phone abuse, the research focuses on sleep interference and its coexistence with using substances such as alcohol and tobacco and with symptomatology and psychiatric comorbidities, particularly anxiety, stress, and depression.

### Interference with Sleep

The sleep interference problem has essentially been observed in adolescence, where in cell-phone abuse can interfere with healthy activities and habits, specifically affecting sleep time and quality. In particular, Sahin et al. (56) observed that the higher students’ points are for problematic use on the Mobile Phone Problem Use Scale (MPPUS) (2), the greater the deterioration in their sleep quality, measured using the Pittsburgh Sleep Quality scale (153).

Along the same lines, Jenaro et al. (69) found that student cell-phone abuse is associated with anxiety and insomnia, particularly in females. Thomée et al. (154, 155) also observed a relationship between the number of calls and messages and sleep difficulties as well as with the tendency to use the phone during the night (59). Similarly, personal stress is thought to be derived from cell-phone abuse insofar as it maintains a state of alertness and interferes with sleep (55).

With respect to social networks, high marks on the BFAS (95) are related to the duration and interruption of sleep during the week, confirming that excessive Facebook use interferes with sleep, decreasing the number of hours slept, and increasing interruptions.

### Substance Use

Substance use in relation to cell phones is often encapsulated within broader research that considers the user’s inability to maintain healthy lifestyle habits, together with symptomatology and psychiatric comorbidities.

In effect, personality problems and psychiatric symptoms coexist with substance and behavioral abuse. If we include the psychological and neurobiological bases of addictions, be they related to substances or behaviors (1, 2, 107, 108, 148, 156, 157), it is natural to observe the coexistence of both, as found in research on the Internet (125). In particular, Lee et al. (158) demonstrated the existence of a neurobiological pattern of common EEG registers for Internet use and depression.

In a study with students, Sanchez Martinez and Otero (74) found a significant relationship between cell-phone abuse, school failure, depressive symptomatology, smoking and consuming cannabis, and other drugs. Similarly, Toda et al. (67) also observed a relationship between cell-phone use and smoking, solely in males, without alcohol consumption, likely due to its lower penetration in their Japanese sample. Social networks have also been shown to coexist with substance use (118).

Therefore, there is a coexistence relationship between substance use and behavioral addiction. In fact, neuroticism predicts the consumption of tobacco, cocaine, and heroin and openness to experience predicts the consumption of marijuana; all of these impulsive behaviors attempt to control internal dysphoric states (128) in a context very similar to cell-phone abuse. However, these types of studies tend to be found within broader research, and there have been few studies specifically focused on the coexistence of problematic cell-phone use and substance use.

### Associated Personality and Psychiatric Problems

Research into psychiatric problems and symptoms is more abundant for the Internet than for cell phones. In the latter, anxiety, depression, and stress are observed, as well as problems with sleep and loneliness. The vast majority of studies have been carried out using students and with diagnostic evaluations that are not always supported by validated or regulated diagnostic instruments.

Augner and Hacker (159) discovered significant relationships between cell-phone abuse, chronic stress, emotional stability, and depression in young women. Tavakolizadeh et al. (84) also observed a coexistence relationship between one’s mental health state – the tendency toward somatization, anxiety, and depression – and excessive cell-phone use.

As previously noted, there are differences between the psychopathological manifestations of problematic cell phone and Internet use, with Internet use demonstrating a majority profile of introversion and loneliness (24). Depression appears to be more consubstantial with Internet use, while anxiety seems to be more consubstantial with problematic cell-phone use, specifically *via* text messaging (79). This indicates that the Internet responds to different psychological behavioral patterns than cell phones.

Social network psychopathological variables tend to be similarly related to the context of the Internet, where problematic use is related to depression and neuroticism, particularly in females (119). The potential differential profile of comorbidities associated with problematic cell-phone use related to applications, such as social networks and instant messaging, needs a thorough revision.

An inverse relationship is apparent between mental health and problematic cell-phone use. In particular, students with lower levels of mental health and psychological stability are more susceptible to developing addictive tendencies to cell phones. These students search for a reduction of tension and dysphoria through social contact, although the existence of manifestations of addiction among healthy students is not excluded with regard to specific or contextual needs (160). Hooper and Zhou (34) indicate, on the contrary, that stress in students with addiction could be the result of problems derived from problematic cell-phone use. Chen (161) also observed a relationship between depression and cell-phone addiction, a coexistence that Young and Rodgers (162) had previously demonstrated, nevertheless indicating that depressive symptoms are associated with many manifestations of alcohol and drug addiction. Therefore, it is unsurprising to find this relationship with respect to the Internet, although it is unknown whether depression points to a vulnerability or consequence.

## Conclusion

We have reviewed problematic cell-phone use with criteria similar to those established for substance addiction or pathological gambling. While we have clearly shown that problematic cell-phone use is an emerging problem that is tightly linked to technological development, there is a lack of coherence and uniformity in the criteria for studying it that requires caution in accepting many of the conclusions indicated.

Undoubtedly, the greatest roadblock to research in cell-phone abuse is the diversity of terms, criteria, and constructs available in the field. Some researchers are convinced that we are facing an addiction unlike any other. In addition, a prudent attitude exists toward the classification of addiction. However, there is an almost indistinguishable or scantly differentiated use of the terms addiction, problematic use, and abuse in the literature. This only adds to the confusion and explains the great diversity of prevalence data in the field and lack of comparability; above all, this diversity of perspectives and lack of conceptual definition has led to studies with very diverse methodologies using samples of convenience, typically consisting of students of very limited size and number of sample points.

In effect, whether or not it is an addiction, cell phones give rise to problems that increasingly affect daily life, for the most part without the risk of uncontrolled spending with the establishment of flat rates or free Wi-Fi access and unlimited use. If we observe the equivalence of its symptoms with the criteria for substance addiction or pathological gambling, a great parallelism is confirmed, corroborated by its coexistence with substance use. We consider that, in effect, we are facing an addiction that is surely not as widespread as some researchers posit. There is a need for a useful conceptualization of the term and a limitation of the boundaries between abuse and addiction and the weight of psychiatric comorbidities, where it is difficult to determine whether problematic use coexists with or is a consequence of them, which becomes more complicated in combined behavioral and substance use addictions.

On the other hand, the majority of the studies have focused on the adolescent and student populations, a period of life where impulsivity and sensation seeking play an important role. Thus, we consider that the concept of cell-phone addiction cannot be extended to the population as a whole until additional data and studies on the adult population are available.

Within the diversity of methodologies, self-reporting is the most frequently used instrument, with all the problems and advantages it entails with respect to the different forms of administration used (mail, email, or phone surveys applied in classes, establishments, street cafés, or university campuses). We know that the context of the application influences a study’s results. Thus, it makes sense to use broad, randomized samples with a controlled context of administration to enable efforts to validate and control the reliability of the questionnaires. Longitudinal studies are novel and are typically completed with cross-sectional questionnaires, but they still suffer from insufficient sample size.

Regarding user profiles, cell-phone use is clearly not an extension of computer use; they are two behaviors with different motivations and user profiles. In both cases, greater impact is found in the young and adolescent population; in the case of the Internet, the users have a wider age range and tend to be more masculine, with a greater presence of introversion and social isolation. Cell-phone abuse, on the contrary, presents a younger, more feminine profile with greater extraversion focused on instant messaging and social networks. Both Internet and cell-phone abuse are associated with problems of self-esteem, self-concept, and neuroticism.

Additional clear identifiers with regards to the problematic cell user profile are lacking. We have previously seen that the data on the socioeconomic levels of the parents and users are not yet consistent. Important cultural and geographic differences are suspected; however, rather than becoming objects of study, these differences have supposed biases that hinder comparability.

With respect to the psychological and psychiatric problems associated with problematic cell-phone use, there is an inverse relationship between mental health, healthy habits, and cell-phone addiction. Comorbidities reported include sleep affectations, anxiety, stress (and depression, to a lesser extent), and consumption of substances, such as alcohol or tobacco, particularly in adolescents. In addition, coexistence with certain psychiatric pathologies, in which lack of impulse control is shared, is also evident.

In summary, there is still much work to be done in this field in light of the limitation of its concepts, criteria, and methodologies. It is highly probable that we may regard the cell phone as an object of easy addiction for vulnerable, addictive, or problematic personalities while allowing for problematic and compulsory use in specific situations and contexts. In addition, it is necessary to broaden the range of analysis in this field to the adult population, with the aim of obtaining a global consideration of the use and abuse of the cell phone. Although the cell phone certainly entails risks for young people and adolescents, problematic consumption indubitably exists in adults as well.

## Author Contributions

Dr. Gabriel Rubio and Dr. Fernando Rodríguez de Fonseca designed the strategy for the present review and selected the topics to be discussed. Prof. José de Sola Gutiérrez searched for the references, read the manuscripts, and wrote the first outline of the review. The three authors reviewed the manuscript and helped with the final writing. Dr. Fernando Rodríguez de Fonseca obtained the financial support.

## Conflict of Interest Statement

The authors declare that the research was conducted in the absence of any commercial or financial relationships that could be construed as a potential conflict of interest.
